# Shoulder pain in primary care: diagnostic accuracy of clinical examination tests for non-traumatic acromioclavicular joint pain

**DOI:** 10.1186/1471-2474-14-156

**Published:** 2013-05-01

**Authors:** Angela Cadogan, Peter McNair, Mark Laslett, Wayne Hing

**Affiliations:** 1 Health and Rehabilitation Research Institute, AUT University, Akoranga Drive, Northcote, Auckland, 1142, New Zealand; 2 Physiotherapy Department, Faculty of Health Sciences and Medicine, Bond University, Gold Coast, Queensland, 4229, Australia

**Keywords:** Sensitivity, Specificity, Shoulder pain, Acromioclavicular joint, Physical examination, Patient history, Local anaesthetic, Primary health care

## Abstract

**Background:**

Despite numerous methodological flaws in previous study designs and the lack of validation in primary care populations, clinical tests for identifying acromioclavicular joint (ACJ) pain are widely utilised without concern for such issues. The aim of this study was to estimate the diagnostic accuracy of traditional ACJ tests and to compare their accuracy with other clinical examination features for identifying a predominant ACJ pain source in a primary care cohort.

**Methods:**

Consecutive patients with shoulder pain were recruited prospectively from primary health care clinics. Following a standardised clinical examination and diagnostic injection into the subacromial bursa, all participants received a fluoroscopically guided diagnostic block of 1% lidocaine hydrochloride (Xylocaine^TM^) into the ACJ. Diagnostic accuracy statistics including sensitivity, specificity, predictive values, positive and negative likelihood ratios (LR+ and LR-) were calculated for traditional ACJ tests (Active Compression/O’Brien’s test, cross-body adduction, localised ACJ tenderness and Hawkins-Kennedy test), and for individual and combinations of clinical examination variables that were associated with a positive anaesthetic response (PAR) (*P*≤0.05) defined as 80% or more reduction in post-injection pain intensity during provocative clinical tests.

**Results:**

Twenty two of 153 participants (14%) reported an 80% PAR. None of the traditional ACJ tests were associated with an 80% PAR (*P*<0.05) and combinations of traditional tests were not able to discriminate between a PAR and a negative anaesthetic response (AUC 0.507; 95% CI: 0.366, 0.647; *P*>0.05). Five clinical examination variables (repetitive mechanism of pain onset, no referred pain below the elbow, thickened or swollen ACJ, no symptom provocation during passive glenohumeral abduction and external rotation) were associated with an 80% PAR (*P*<0.05) and demonstrated an ability to accurately discriminate between an PAR and NAR (AUC 0.791; 95% CI 0.702, 0.880; *P*<0.001). Less than two positive clinical features resulted in 96% sensitivity (95% CI 0.78, 0.99) and a LR- 0.09 (95% CI 0.02, 0.41) and four positive clinical features resulted in 95% specificity (95% CI 0.90, 0.98) and a LR+ of 4.98 (95% CI 1.69, 13.84).

**Conclusions:**

In this cohort of primary care patients with predominantly subacute or chronic ACJ pain of non-traumatic onset, traditional ACJ tests were of limited diagnostic value. Combinations of other history and physical examination findings were able to more accurately identify injection-confirmed ACJ pain in this cohort.

## Background

Disorders of the acromioclavicular joint (ACJ) are a common cause of shoulder pain in primary care, affecting patients of all ages and levels of activity [1]. Acromioclavicular joint pain has many causes including capsulo-ligamentous injury and instability [2-4], degenerative or post-traumatic arthropathy, inflammatory arthropathy [5], crystal arthropathy and osteolysis [6].

The clinical diagnosis of painful ACJ disorders is important to enable efficient application of appropriate treatment interventions and to inform decisions regarding the need for further diagnostic investigations or specialist consultation. The clinical detection of painful ACJ conditions also aids interpretation of the relevance of abnormal radiological findings, the prevalence of which is known to be high in asymptomatic individuals [7]. Detection of painful ACJ conditions is also of prognostic significance having been identified as one of the strongest factors associated with reduced patient satisfaction [8] and with reduced functional ability following rotator cuff repair surgery [9].

While debate surrounds the accuracy of clinical tests for the diagnosis for shoulder conditions such as subacromial impingement [10,11] and glenoid labrum tears [12], the clinical diagnosis of ACJ pain is considered to be less contentious with localised ACJ tenderness [13], the O’Brien’s/Active Compression test [13-15], the cross-body adduction test [14,16] and the Hawkins-Kennedy [13,14] frequently reported in diagnostic studies as index tests for identifying injection-confirmed ACJ pain (Table 1). Despite moderate to high sensitivity and specificity values for these tests, several methodological considerations may influence the interpretation and subsequent application of these results in the primary care setting. Firstly, all patients in these studies were sampled from either surgical settings [14-16] or from a specialist shoulder referral centre [13]. The mechanism of injury as well as the prevalence of painful ACJ conditions and the severity of ACJ disease in these settings are likely to differ considerably from patients presenting with shoulder pain in primary health care. Differences in prevalence (pre-test probability) and disease severity (spectrum bias) are known to affect the performance of diagnostic tests across populations in which these characteristics differ [17] and the direct application of diagnostic accuracy probabilities obtained for ACJ tests in surgical care settings to primary health care settings may lead the clinician to an incorrect diagnostic conclusion potentially resulting in inappropriate management pathways.

**Table 1:** Summary of previous studies investigating the diagnostic accuracy of clinical tests for acromioclavicular joint pain and pathology

**Test**	**Author and Year**	**Recruitment**	**Sensitivity**	**Specificity**	**LR+**	**LR-**	**QUADAS Score**^**a**^
Active Compression/O’Brien’s test	O’Brien et al. 1998 [15]	Orthopaedic hospital	93	96	23.1	0.08	5
	Walton et al. 2004 [13]	Specialist shoulder referral centre	16	90	1.6	0.93	13
	Chronopoulos et al. 2004 [14]	Orthopaedic surgical waiting list	41	95	8.2	0.62	10
	Van Riet & Bell 2011 [16]	Orthopaedic hospital	83	N/A	N/A		
Cross-body adduction	Chronopoulos et al. 2004 [14]	Orthopaedic surgical waiting list	77	79	3.67	0.29	10
	Van Riet & Bell 2011 [16]	Orthopaedic hospital	67	N/A	N/A	N/R	
Localised ACJ tenderness	Walton et al. 2004 [13]	Specialist shoulder referral centre	96	10	1.07	0.40	13
Hawkins Kennedy test	Chronopoulos et al. 2004 [14]	Orthopaedic surgical waiting list	47	45	0.85	1.2	10

*Abbreviations:* LR+, positive likelihood ratio; LR-, negative likelihood ratio; QUADAS, Quality Assessment of Diagnostic Accuracy Studies tool [45]; N/A, not applicable; N/R, not reported; ACJ, acromioclavicular joint.

^a^From Hegedus et al. (2008) [30].

In addition, many of the studies [14-16] contained several sources of bias that may influence the interpretation of the findings [18]. In two previous studies that reported the highest levels of diagnostic accuracy for ACJ pain, blinding procedures were not reported [14,15]. The reference standard used for identification of ACJ pain in previous studies also varied or was inconsistently applied. Partial verification bias, incorporation bias and selection bias were present in several studies [14-16] and the positive anaesthetic response criteria varied in all studies, ranging from >50% pain relief [13], “complete or near complete relief” [14], or “all ACJ clinical tests had to be negative following injection” [16], making it difficult to compare results between studies. These sources of bias may result in under- or overestimation of diagnostic test performance [19].

The lack of diagnostic studies in primary care populations and the methodological concerns from previous studies conducted in secondary care settings mean the accuracy of clinical tests for identifying ACJ pain and pathology in the primary care population is unknown and previous results may be incorrectly interpreted or wrongly applied. Hence, the purpose of this study was to estimate the diagnostic accuracy of traditional ACJ tests in a primary care population and compare their accuracy with other clinical examination findings to determine the most valid tests for identifying the ACJ as the predominant source of pain in a primary care setting.

## Methods

### Study design and setting

The study was designed using the STARD guideline principles [20]. Participants were recruited from community-based medical and physiotherapy practices across Christchurch, New Zealand. Ethical approval was granted by the New Zealand Ministry of Health Regional Ethics Committee (Upper South A) and written informed consent was provided by all participants prior to participation in the study.

### Recruitment and sampling

**Figure 1:**
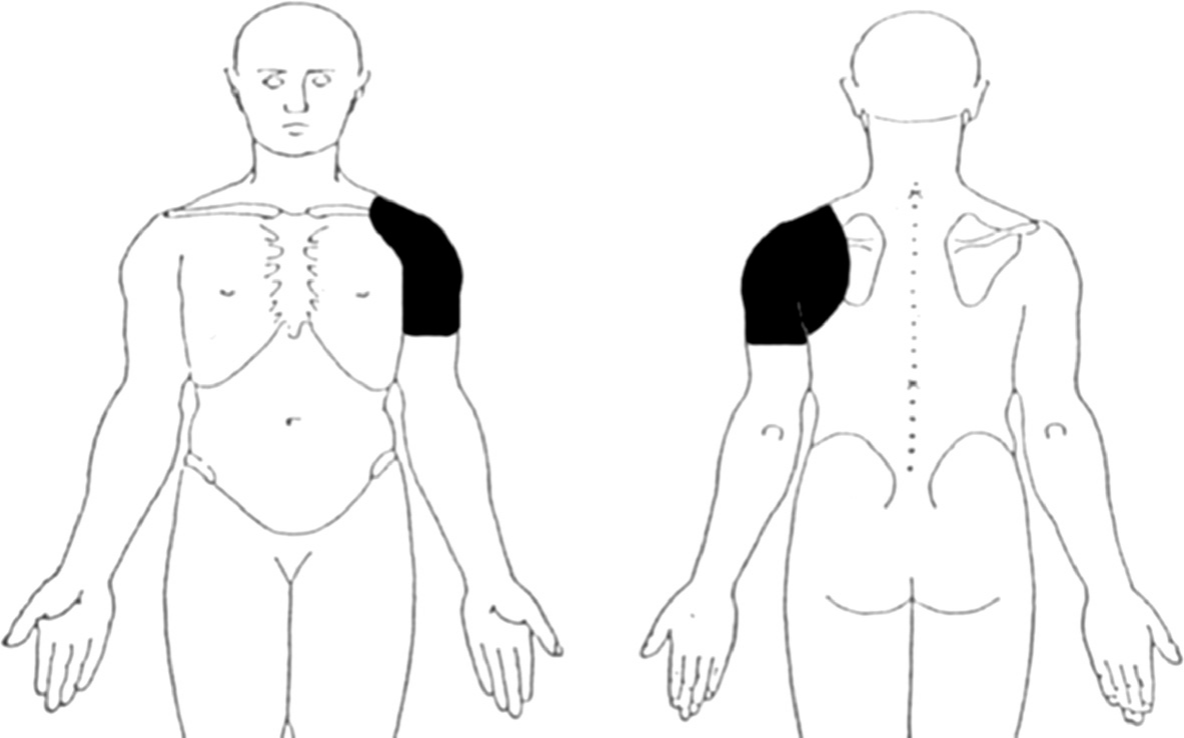
Location of primary shoulder pain required for inclusion in the study.

Consecutive patients over the age of 18 years, presenting to their primary care practitioner (general practitioner or physiotherapist) for the first time with a new episode of shoulder pain (Figure 1) and with the ability to follow verbal instructions, were eligible for inclusion in the study. Exclusion criteria were known fractures or dislocations around the shoulder complex, referred pain from the cervical spine, sensory or motor deficit involving the upper limb, previous surgery to the shoulder or cervical spine, or contraindications to imaging or injection procedures. Sample size was determined using methods for estimates for diagnostic accuracy studies described by Flahault et al. [21]. The minimum acceptable lower confidence limit was set at 0.75 and expected sensitivity/specificity were both set at 0.90.

### Index tests

All participants completed self-report questionnaires including the Shoulder Pain and Disability Index (SPADI) [22], Global Disability Rating [23], SF-8^TM^ health survey [24], and Fear Avoidance Beliefs Questionnaire (FABQ) [25]. All history and physical examination variables were selected according to evidence or clinical relevance for subacromial and ACJ pathology. A full list of clinical examination variables is presented in Additional file 1. All clinical examinations were conducted by a musculoskeletal physiotherapist with 20 years’ experience.

All participants recorded a standardised history including medical and family history, smoking history, a pain drawing, symptom duration and details of past history of shoulder pain, occupational, sporting and recreational activities. Details of the mechanism of injury were also recorded and coded as traumatic (external force, fall or impact), strain (intrinsic stretch, reach or lifting injury), repetitive (onset of pain during or within 48 hours of repetitive activity for which no other cause was identified), or unknown.

The physical examination consisted of active range of motion (ROM) of the cervical spine [26], inspection for swelling or muscle atrophy, recording the presence of a painful arc of motion during abduction [27], recording of symptom responses associated with arm elevation (flexion), scapuloclavicular tests [28], passive ROM glenohumeral abduction, external rotation performed at 0° abduction and internal and external rotation performed at 90° of abduction [29], cross-body adduction, isometric resisted muscle tests (abduction, external and internal rotation), orthopaedic tests selected according to evidence for reported diagnostic accuracy [30] and performed as described by the original authors; Hawkins-Kennedy test [31], active compression (O’Brien’s) test [15], and pain responses to palpation of the ACJ. Symptom responses were recorded during all ROM and resisted tests according to whether or not typical symptoms were reproduced.

**Figure 2:**
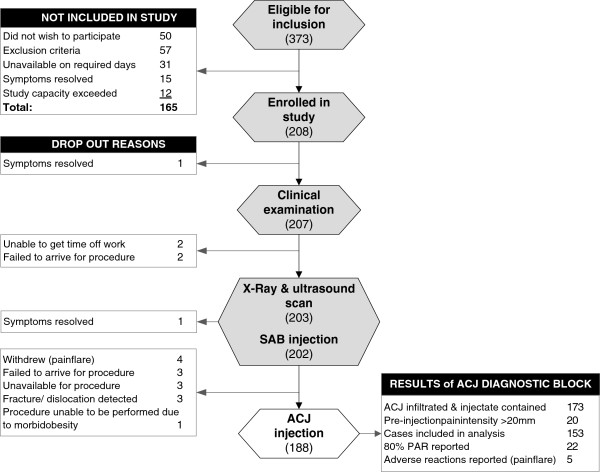
**Flow chart of study procedures, drop out explanations and adverse events.***Abbreviations:* SAB, subacromial bursa; ACJ, acromioclavicular joint; PAR, positive anaesthetic response.

During the physical examination, those tests provocative of typical pain were identified for use in pre- and post-injection testing. Ambiguous or indeterminate results of clinical examination tests were recorded and coded as missing data. Following the clinical examination, all participants received a standardised shoulder x-ray series, diagnostic ultrasound scan, and subacromial bursa diagnostic block as part of the larger diagnostic accuracy study (Figure 2) [32].

### Reference standard test

One week following the imaging investigations and subacromial bursa injection, participants received a fluoroscopically guided injection of local anaesthetic into the ACJ. Participants were positioned supine with the arm in external rotation. Under aseptic conditions, a 22-gauge needle was inserted into the ACJ using a direct anterior approach. Iodinated contrast (0.5 ml of Omnipaque 300 GE Healthcare) was introduced and fluoroscopic images used to confirm needle placement within the ACJ. Approximately 2 mL of 1% lidocaine hydrochloride (Xylocaine^TM^) was then injected into the joint. The radiologist recorded whether the ACJ was successfully infiltrated and whether the injectate was contained within the joint. This procedure has been described in detail elsewhere [32].

Immediately prior to the injection, all participants were examined using up to six tests identified during the clinical examination as being provocative of typical symptoms. Pre-injection pain intensity was recorded for each clinical test on a 100 mm visual analogue scale (VAS; 0 mm “no pain” and 100 mm “worst imaginable pain”). Tests were repeated between 5 and 15 minutes following the injection and pain intensity scores recorded again. Percent change in pain intensity was calculated for each index test and the average change in pain intensity from all clinical tests was calculated. Positive integers (+) indicate increased post-injection pain intensity, and negative integers (−) indicate decreased post-injection pain intensity.

A positive anaesthetic response (PAR) was set at 80% or more reduction in post-injection pain intensity. Higher PAR cut-points have been shown to reduce the false-positive response rate in patients with confounding factors reporting spinal pain [33] and to produce high specificity with regard to identification of the tissue origin of pain [34]. The 80% PAR criteria has also shown diagnostic stability at 2-year follow-up compared with lower PAR cut-points [35] and is now considered the criterion standard for the selection of high quality studies for inclusion in systematic reviews of diagnostic efficacy in spinal conditions in which the use of diagnostic injections is well established [33].

The investigator performing the clinical examination and pre- and post-injection clinical tests was blinded to any diagnostic or treatment information from referring practitioners and to imaging results. Radiologists were not provided with any clinical information prior to the imaging or injection procedures.

### Statistical analysis

Sensitivity, specificity, positive and negative predictive values, positive likelihood ratios (LR+), negative likelihood ratios (LR-) were calculated for individual, and combinations of traditional ACJ tests (Active Compression/O’Brien’s test, cross-body adduction, ACJ palpation and Hawkins-Kennedy test) using Confidence Interval Analysis (CIA) software (version 2.1.2) [36]. Likelihood ratios were interpreted according to reported guidelines for interpreting changes in probability of disease status (positive likelihood ratio: small (2.0 to 5.0), moderate (5.0 to 10.0), large (>10); negative likelihood ratio: small (0.2 to 0.5), moderate (0.1 to 0.2), large (<0.1)) [37]. Area under the receiver operator curve with 95% confidence intervals (CI) was calculated for combinations of traditional tests using the Statistical Package for the Social Sciences (SPSS version 17.0, IBM® Corporation 2010).

The Fisher exact test was performed on all clinical examination variables (Additional file 1) using the Statistical Package for the Social Sciences (SPSS version 17.0, IBM® Corporation 2010). The clinical examination variables that demonstrated the strongest association with an 80% PAR to ACJ diagnostic block were identified (*P*≤ .0.05) and the diagnostic accuracy estimates for these clinical examination variables was calculated as described above [36]. The diagnostic accuracy estimates for these clinical features were then compared with the estimates for traditional ACJ tests to determine the individual or combinations of clinical features that were most accurate for identifying an 80% PAR following ACJ diagnostic injection.

## Results

Three hundred and seventy three patients were referred to the study between July 2009 and June 2010 resulting in 208 participants being included in the study. Reasons for exclusion of patients in the study are presented in Figure 2. There were no significant differences between those included and excluded from the study with respect to age or gender. Those excluded from the study reported shorter duration of symptoms (median 2 weeks; IQ range 4 weeks) (Mann–Whitney *P<*0.001).

Two hundred and seven subjects completed the clinical examination and 188 participants received the ACJ diagnostic block. Demographic data for those who underwent the ACJ diagnostic block are presented in Table 2. A non-traumatic mechanism of injury was reported by 62% of all participants (Table 2). Drop-out explanations are presented in Figure 2. There were no differences in demographic or self-report questionnaire results between those who completed the study and those who dropped out (*P*>0.05). Mean time between the clinical examination and ACJ diagnostic block was 11 days (± 3 days), range 8 to 19 days.

**Table 2:** Demographic information

**Participant characteristics**	**All participants (N=188)**		**PAR group (n=22)**	**NAR group (n=131)**
	**Mean (SD)**	**Range**	**Mean (SD)**	**Mean (SD)**
Age (years)	42 (13)	18 - 81	41 (13)	43 (14)
Height (cm)	172 (10)	147 - 199	170 (11)	172 (10)
Weight (kg)	80.4 (16.7)	50.3 – 135.4	78.5 (16.1)	80.4 (17.0)
Symptom duration (weeks)^†^	7 (14)^†^	0 – 175	6 (18)^†^	8 (14)^†^
Worst pain in last 48 hrs (100mm VAS scale)	62 (23)	3 – 100	59 (17)	65 (22)
SF8 physical component score (%)	44 (8)	23 – 61	45 (7)	44 (8)
SF8 mental component score (%)^†^	54 (11)^†^	27 – 66	57 (11)^†^	54 (10)^†^
SPADI pain score (%)	50 (22)	0 – 100	49 (15)	51 (21)
SPADI disability score (%)^†^	26 (30)^†^	0 – 96	26 (21)^†^	28 (30)^†^
SPADI total (%)	37 (20)	0 – 98	35 (13)	38 (21)
FABQ physical activity score (%)	65 (22)	0 – 100	64 (20)	65 (67)
FABQ work score (%)^a†^	21 (44)^†^	0 – 81	32 (47)^†^	21 (44)^†^
FABQ total score (%)^a^	41 (19)	0 – 87	45 (18)	41 (18)
% male gender	52		55	56
% right hand dominant	87		86	87
% dominant arm affected	52		36	55
% Pain onset: traumatic	38		32	40
% Pain onset: non-traumatic	62		68	61
% Pain onset: strain injury	40		36	41
% Pain onset: repetitive activity	13		27	10*
% Pain onset: insidious	9		5	10

*Abbreviations*. PAR, positive anaesthetic response (≥80% post-injection reduction in pain intensity); NAR, negative anaesthetic response (<80% reduction in post-injection pain intensity); VAS, 100 mm visual analogue pain score in previous 48 hours; SPADI, Shoulder Pain & Disability Index; FABQ, Fear Avoidance Beliefs Questionnaire.

^a^only cases ‘in paid employment’ used in analysis.

^†^Variables were not normally distributed. Median (interquartile range) values are presented.

*P<0.05.

### Reference standard procedure

Average ACJ injection volume was 2.1 mL (SD 0.7 mL), and the injectate was contained within the ACJ in 174 cases (93%). Due to the known limitations of VAS scales for measuring change in pain intensity when pre-injection pain levels are low (<20 mm) [38], only cases where pre-injection pain intensity exceeded 20 mm were included in the analysis of anaesthetic response to diagnostic injections (n=153). A PAR (≥80% reduction in post-injection pain intensity) was reported by 22 of the 153 participants (14%). The distribution of pathology identified on diagnostic imaging investigations for the PAR and negative anaesthetic response (NAR) groups is presented in Table 3.

**Table 3:** Distribution of diagnostic imaging results

**Diagnostic test results**	**Total identified (N=153)**	**% in PAR group with pathology (n=22)**	**% in NAR group with pathology (n=131)**
	**n (%)**	**%**	**%**
**X-ray**			
ACJ pathology	21 (14)	23	12
ACJ arthropathy	18 (12)	18	11
ACJ osteolysis	6 (4)	5	4
GHJ pathology	7 (5)	0	5
Rotator cuff calcification	19 (12)	5	14
supraspinatus	11 (7)	5	8
infraspinatus	7 (5)	0	5
subscapularis	6 (4)	0	5
**Ultrasound**			
SAB pathology	105 (69)	55	71
Rotator cuff tear	46 (30)	14	33
supraspinatus	38 (25)	14	27
infraspinatus	3 (2)	0	2
subscapularis	10 (7)	0	8
Rotator cuff tendinosis	21 (14)	9	15
supraspinatus	20 (13)	9	14
infraspinatus	1 (1)	0	1
subscapularis	1 (1)	0	1
Rotator cuff calcification	35 (23)	18	24
supraspinatus	22 (14)	18	14
infraspinatus	9 (6)	0	7
subscapularis	15 (10)	5	11
LHB tear or tendinosis	6 (4)	5	4
Biceps tendon sheath effusion	21 (14)	9	15
ACJ pathology	35 (23)	41	20
GHJ effusion	6 (4)	0	5

*Abbreviations.* ACJ, acromioclavicular joint; PAR, positive anaesthetic response (≥80% post-injection reduction in pain intensity); NAR, negative anaesthetic response (<80% reduction in post-injection pain intensity); GHJ, glenohumeral joint; SAB, subacromial bursa; LHB, long head of biceps.

*Note:* There were no statistically significant differences in frequency of imaging findings between the PAR and NAR groups (*P*>0.05).

### Accuracy of traditional acromioclavicular joint tests

The diagnostic accuracy results for traditional ACJ tests are presented in Table 4. No traditional ACJ tests were associated with a PAR following ACJ diagnostic block (*P*<0.05). Sensitivity for traditional tests ranged from 0.14 (95% CI 0.05, 0.33) (Active Compression/O’Brien’s test) to 0.70 (95% CI 0.48, 0.86) (Hawkins-Kennedy test). Specificity ranged from 0.26 (95% CI 0.19, 0.35) (cross-body adduction test) to 0.92 (95% CI 0.86, 0.96) (Active Compression/O’Brien’s test). Positive likelihood ratios ranged from 0.86 (cross-body adduction) to 1.73 (Active Compression/O’Brien’s test) and negative likelihood ratios ranged from 0.84 (Hawkins-Kennedy test) to 1.39 (cross-body adduction). Positive predictive values (PPV) ranged from 0.13 to 0.23, with the largest change in post-test probability (positive predictive value) for a PAR observed for the Active Compression/O’Brien’s test (0.23; 95% CI 0.08, 0.50) based upon a pre-test probability (prevalence) of 0.14 (14%).

**Table 4:** Diagnostic accuracy of traditional tests for acromioclavicular joint pain

**Clinical tests**	**Cell counts**				**Diagnostic accuracy**						
	**TP**	**FN**	**FP**	**TN**	**Sensitivity (95% CI)**	**Specificity (95% CI)**	**PPV (95% CI)**	**NPV (95% CI)**	**LR+ (95% CI)**	**LR- (95% CI)**	**OR (95% CI)**
***Individual tests***											
Cross-body adduction	14	8	93	33	0.64 (0.43, 0.80)	0.26 (0.19, 0.35)	0.13 (0.08, 0.21)	0.81 (0.66, 0.90)	0.86 (0.58, 1.12)	1.39 (0.71, 2.43)	0.62 (0.24, 1.61)
Active Compression/O’Brien’s test	3	19	10	*117*	0.14 (0.05, 0.33)	0.92 (0.86, 0.96)	0.23 (0.08, 0.50)	0.86 (0.79, 0.91)	1.73 (0.53, 5.15)	0.94 (0.72, 1.06)	1.85 (0.47. 7.33)
Hawkins-Kennedy test	14	6	81	45	0.70 (0.48, 0.86)	0.36 (0.28, 0.44)	0.15 (0.09, 0.23)	0.88 (0.77, 0.95)	1.09 (0.74, 1.41)	0.84 (0.39, 1.55)	1.30 (0.47, 3.61)
Localised ACJ tenderness	8	14	34	94	0.36 (0.20, 0.57)	0.73 (0.65, 0.80)	0.19 (0.10, 0.33)	0.87 (0.79, 0.92)	1.37 (0.70, 2.39)	0.87 (0.58, 1.13)	1.58 (0.61, 4.10)
***Combinations of tests***											
At least 1 of 4	21	1	120	9	0.96 (0.78, 0.99)	0.07 (0.04, 0.13)	0.15 (0.10, 0.22)	0.90 (0.60, 0.98)	1.03 (0.84, 1.11)	0.65 (0.11, 3.54)	1.58 (0.19, 13.09)
At least 2 of 4	11	9	77	43	0.55 (0.34, 0.74)	0.36 (0.28, 0.45)	0.13 (0.07, 0.21)	0.83 (0.70, 0.91)	0.86 (0.53, 1.20)	1.26 (0.69, 2.01)	0.68 (0.26, 1.78)
At least 3 of 4	6	14	22	93	0.30 (0.15, 0.52)	0.81 (0.73, 0.87)	0.21 (0.10, 0.40)	0.87 (0.79, 0.92)	1.57 (0.70, 3.13)	0.87 (0.59, 1.09)	1.81 (0.63, 5.25)
4 of 4	1	19	1	113	0.05 (0.01, 0.24)	0.99 (0.95, 1.00)	0.50 (0.10, 0.91)	0.86 (0.79, 0.91)	5.70 (0.60, 52.63)	0.96 (0.77, 1.01)	5.95 (0.36, 99.19)

*Abbreviations.* TP, true positives; FN, false negatives; FP, false positives; TN, true negatives; CI, confidence interval; PPV, positive predictive value; NPV, negative predictive value; LR+, positive likelihood ratio; LR-, negative likelihood ratio; OR, odds ratio.

*Note:* Cell counts do not total 153 in some cases due to missing data.

All *P*-values for the OR were >0.05 (not significant).

When the traditional tests were combined using minimum numbers of positive tests, sensitivity was highest (0.96; 95% CI 0.78, 0.99) when none of the tests were positive and specificity was highest (0.99; 95% CI 0.95, 1.00) when all four tests were positive. The highest positive likelihood was 5.70 (95% CI 0.60, 52.63) (four positive tests) and the lowest negative likelihood ratio was 0.65 (95% CI 0.11, 3.54) (minimum of one positive test). The largest change in post-test probability of a PAR was observed when 4 tests were positive (PPV 0.50; 95% CI 0.10, 0.91). For the traditional ACJ tests, the area under the receiver operator curve for the total number of positive tests was 0.507 (*P*=0.920; 95% CI: 0.366, 0.647).

### Index tests

Five clinical variables were identified from the standardised history and physical examination that were associated with an 80% PAR following ACJ diagnostic injection (*P*≤0.05) (Table 5): repetitive mechanism of pain onset; the absence of referred pain below the elbow; visual observation of a thickened or swollen ACJ; typical symptoms were not reproduced or aggravated by passive GHJ abduction; typical symptoms were not reproduced or aggravated by passive GHJ external rotation (performed at 90° abduction). Missing data for these variables was less than 5%.

**Table 5:** Diagnostic accuracy of individual history and physical examination variables for a positive response to acromioclavicular joint diagnostic block

**Clinical examination variables**	**Cell counts**				**Diagnostic accuracy**						
	**TP**	**FN**	**FP**	**TN**	**Sensitivity(95% CI)**	**Specificity(95% CI)**	**PPV (95% CI)**	**NPV (95% CI)**	**LR+ (95% CI)**	**LR- (95% CI)**	**OR (95% CI)**
Onset: repetitive activity	6	16	13	118	0.27 (0.13, 0.48)	0.90 (0.84, 0.94)	0.32 (0.15, 0.54)	0.88 (0.82, 0.93)	2.75 (1.15, 6.07)	0.81 (0.57, 0.98)	3.4* (1.1, 10.2)
No pain referred below elbow	20	0	105	23	1.00 (0.84, 1.00)	0.18 (0.12, 0.26)	0.16 (0.11, 0.23)	1.00 (0.86, 1.00)	1.22 (1.18, 1.34)	0.00 (0.00, 0.92)	0.84* (0.78, 0.91)
ACJ thickened or swollen	15	5	47	77	0.75 (0.53, 0.89)	0.62 (0.53, 0.70)	0.24 (0.15, 0.36)	0.94 (0.87, 0.97)	1.98 (1.33, 2.70)	0.40 (0.28, 0.77)	4.9** (1.7, 14.4)
PROM GHJ abduction – no pain	8	14	18	108	0.36 (0.20, 0.57)	0.86 (0.79, 0.91)	0.31 (0.17, 0.50)	0.89 (0.82, 0.93)	2.55 (1.23, 4.86)	0.74 (0.50, 0.95)	3.4* (1.3, 9.3)
PROM ER90^0^ – no pain	11	11	23	107	0.50 (0.31, 0.69)	0.82 (0.75, 0.88)	0.32 (0.19, 0.49)	0.91 (0.84, 0.95)	2.83 (1.56, 4.76)	0.61 (0.37, 0.85)	4.7** (1.8, 12.0)

*Abbreviations.* TP, true positives; FN, false negatives; FP, false positives; TN, true negatives; CI, confidence interval; PPV, positive predictive value; NPV, negative predictive value; LR+, positive likelihood ratio; LR-, negative likelihood ratio; OR, odds ratio; ACJ, acromioclavicular joint; PROM, passive range of motion; GHJ, glenohumeral joint; ER 90°, external rotation performed in 90° of abduction.

*Note.* Cell counts do not total 153 in some cases due to missing data.

*P<0.05; ** *P<*0.01.

Sensitivity estimates for these clinical variables ranged from 0.27 (repetitive mechanism of injury; 95% CI 0.13, 0.48) to 1.00 (no referred pain below the elbow; 95% CI 0.84, 1.00). Specificity ranged from 0.18 (no referred pain below the elbow; 95% CI 0.12, 0.26) to 0.90 (repetitive mechanism of injury; 95% CI 0.84, 0.94). The highest positive likelihood ratio was recorded when passive external rotation performed at 90° abduction did not reproduce typical symptoms (2.83; 95% CI 1.56, 4.76) and the lowest negative likelihood ratio for an 80% PAR occurred when pain did not refer below the elbow (0.00; 95% CI 0.00, 0.92). Compared with the pre-test probability of 14%, the largest change in post-test probability (positive predictive value) of a PAR (0.32) was observed for pain onset due to repetitive activity (95% CI 15, 54) and the absence of pain during passive external rotation performed at 90° abduction (95% CI 0.19, 0.49).

**Figure 3:**
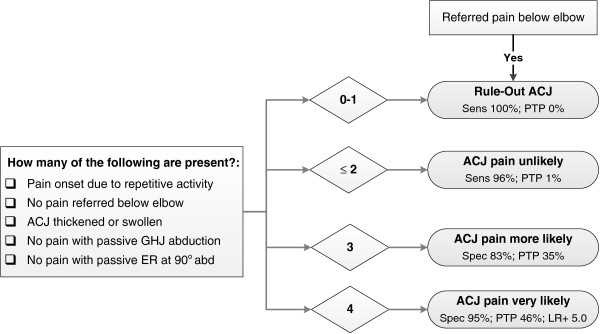
**Diagnostic decision guideline for identifying pain arising from the acromioclavicular joint.** Clinical tests found to be of most diagnostic value for identifying a predominant acromioclavicular joint pain source defined by ≥80% pain relief following injection of local anaesthetic into the acromioclavicular joint. *Abbreviations:* ACJ, acromioclavicular joint; sens, sensitivity; PTP, post-test probability of ACJ pain at the 80% pain reduction standard, based upon pre-test probability (prevalence) of 14%; HBB, hand-behind-back; GHJ, glenohumeral joint; ER, external rotation; abd, abduction; spec, specificity.

When the history and physical examination findings were combined, highest sensitivity (1.00; 95% CI 0.85, 1.00) and lowest LR- (0.00; 95% CI 0.00, 2.15) were observed when none of the findings were positive, and highest specificity (0.95; 95% CI 0.90, 0.98) and LR+ (4.98; 95% CI 1.69, 13.84) occurred when four or more tests were positive (Table 6). None of the participants recorded positive responses for all five clinical features. Compared with a pre-test probability of 14%, the largest change in post-test probability (positive predictive value) for a PAR was observed when at least four of the five tests were positive (0.46). Area under the receiver operator curve for the total number of positive clinical tests was 0.791 (*P<*0.001; 95% CI: 0.702, 0.880). These diagnostic accuracy results have been summarized into a guideline that may be used to assist diagnostic decision making in clinical practice (Figure 3).

**Table 6:** Diagnostic accuracy of combinations of history and physical examination variables for a positive response to acromioclavicular joint diagnostic block

**Number of positive clinical tests**^**a**^	**Cell counts**				**Diagnostic accuracy**						
	**TP**	**FN**	**FP**	**TN**	**Sensitivity (95% CI)**	**Specificity (95% CI)**	**PPV (95% CI)**	**NPV (95% CI)**	**LR+ (95% CI)**	**LR- (95% CI)**	**OR (95% CI)**
One or more	22	0	116	9	1.00 (0.85, 1.00)	0.07 (0.04, 0.13)	0.16 (0.11, 0.23)	1.00 (0.70, 1.00)	1.08 (1.07, 1.15)	0.00 (0.00, 2.15)	0.84 (0.78, 0.90)
Two or more	21	1	61	70	0.96 (0.78, 0.99)	0.53 (0.45, 0.62)	0.26 (0.17, 0.36)	0.99 (0.92, 1.00)	2.05 (1.61, 2.52)	0.09 (0.02, 0.41)	24.10*** (3.15, 184.45)
Three or more	12	10	22	109	0.55 (0.35, 0.73)	0.83 (0.76, 0.89)	0.35 (0.22, 0.52)	0.92 (0.85, 0.95)	3.25 (1.83, 5.40)	0.55 (0.32, 0.79)	5.95*** (2.29, 15.47)
Four or more	5	17	6	125	0.23 (0.10, 0.43)	0.95 (0.90, 0.98)	0.46 (0.21, 0.72)	0.88 (0.82, 0.92)	4.98 (1.69, 13.84)	0.81 (0.59, 0.95)	6.13** (1.69, 22.27)
Five or more	0	22	0	131	†	†	†	†	†	†	†

*Abbreviations.* TP, true positives; FN, false negatives; FP, false positives; TN, true negatives; CI, confidence interval; PPV, positive predictive value; NPV, negative predictive value; LR+, positive likelihood ratio; LR-, negative likelihood ratio; OR, odds ratio.

*Note:* Cell counts do not total 153 in some cases due to missing data.

^a^Any combination of the following positive tests: onset of pain due to repetitive activity; no pain referral below elbow; ACJ thickened or swollen; typical symptoms are not provoked during PROM GHJ abduction or PROM external rotation (90° abduction).

† Invalid calculation (no participants reported five positive clinical tests).

** *P*≤0.01; *** *P*≤0.001.

## Discussion

These results provide preliminary evidence that traditional ACJ tests considered to be diagnostic for ACJ pain were of limited diagnostic value in a cohort of primary care patients with predominantly subacute or chronic ACJ pain of non-traumatic onset. Combinations of other history and physical examination findings were able to more accurately identify ACJ pain in this cohort.

Results of this study represent predominantly non-traumatic ACJ pain. For the purposes of this study, trauma was defined as an involving an ‘external force, impact or fall’, and 62% of participants reported that the onset of their pain was not associated with a traumatic event. Due to the nature of the reference standard procedure used in this study (intra-articular ACJ diagnostic injection), a valid reference standard outcome required an intact joint capsule to minimise the possibility of anaesthetisation of extra-articular ACJ structures that may produce a false-positive result. For this reason, frank ACJ instabilities such as subluxations or dislocations were excluded from our study. It is possible that participants with more subtle ACJ capsulo-ligamentous injuries were included in the study, but the subsequent use of fluoroscopy during the injection procedure enabled exclusion from analysis those cases in which the joint capsule was compromised, evidenced by the injectate breaching the ACJ capsular margins. For these reasons, our results reflect patients in whom the ACJ capsulo-ligamentous complex is largely intact, and cannot be generalised to those patients with traumatic ACJ capsulo-ligamentous disruption.

The highest levels of diagnostic accuracy for identifying a predominant ACJ pain source in this primary care study were observed for combinations of five history and physical examination variables: repetitive mechanism of pain onset, absence of referred pain below the elbow, a thickened or swollen ACJ and the absence of typical pain provocation during passive GHJ abduction and external rotation (performed at 90° abduction). The area under the receiver operator curve (AUC) indicated that combinations of these five clinical features were considerably more accurate in identifying those likely to report an 80% PAR (predominant ACJ pain source) than traditional ACJ tests. For those participants who reported less than two of the five positive clinical features, the likelihood of an 80% PAR (predominant ACJ pain source) was very low (LR- 0.09; 95% CI 0.02, 0.41). Combined with high sensitivity (0.96; 95% CI 0.78, 0.99), the presence of less than two positive clinical features may assist in ruling-out a predominant ACJ pain source with moderate to high levels of confidence.

Combinations of these clinical features were also of value for positively identifying those who were likely to report an 80% PAR (predominant ACJ pain source). When four positive clinical features were identified, participants were almost five times more likely to report a PAR (LR+ 4.98; 95% CI 1.69, 13.84) than those for whom less than four clinical features were identified. Specificity was also high (0.95; 95% CI 0.90, 0.98) indicating a low false positive rate when four of the five clinical features were present. These results support previous findings of improved diagnostic accuracy using combinations of clinical examination findings, compared with individual physical examination tests for painful ACJ conditions [14]. Figure 3 presents a diagnostic decision guideline that may aid utilization of these results in clinical practice.

This study identified several history and physical examination features that, to our knowledge, have not previously been reported as diagnostically useful for ACJ pain. The onset of pain due to repetitive activity demonstrated high specificity in our study (0.90; 95% CI 0.84, 0.94). Other literary references to repetitive injury mechanisms in the diagnosis of shoulder pain relate to osteolysis of the distal clavicle that causes pain in the region of the ACJ [39,40]. Although 24 participants reported the onset of pain related to repetitive activity in our study, there were only six cases in which the radiographic finding of osteolysis was reported. Hence, while the high specificity suggests this finding may help to rule-in ACJ pain, other painful shoulder conditions may also result from, or become aggravated by repetitive activity and this finding must be interpreted with caution with respect to assumptions regarding the underlying pathology.

The relationship between the absence of pain during passive glenohumeral joint abduction and external rotation (performed at 90° abduction) and ACJ pain were new and potentially important findings. The provocation of pain during passive glenohumeral joint abduction and external rotation is commonly associated with symptomatic glenohumeral capsuloligamentous or intra-articular pathology [41-43]. Despite modest LR+ (2.6 and 2.8 respectively), the absence of symptom provocation during GHJ abduction and external rotation resulted in the largest improvement in the post-test probability of an 80% PAR (predominant ACJ pain source) of all traditional ACJ tests and other clinical examination features investigated in this study. Given the diverse nature of shoulder pain seen in primary care, and the lack of specificity of clinical tests for many shoulder conditions, the exclusion of diagnostic possibilities with the help of ‘negative tests’ may assist in narrowing the range of differential diagnoses representing a more realistic approach to diagnostic reasoning for the shoulder than the pursuit of the ‘magic bullet’ test with high specificity for shoulder disorders.

### Performance of traditional acromioclavicular joint tests

Traditional ACJ tests were of limited diagnostic value for identifying those with a predominant ACJ pain source in this primary care cohort. Of the traditional tests, the positive likelihood ratio of the Active Compression/O’Brien’s test was only 1.73, with a lower 95% confidence limit of 0.53, reducing confidence that a positive test improved the likelihood of a PAR in individual patients above the level of ‘chance’ (1.0). These results are similar to those reported by Walton et al. (2004) (LR+ 1.6) in a small cohort of patients (n=38) referred to a specialist shoulder centre [13]. Other authors, including those who developed the test [15] have reported larger positive likelihood ratio values for the Active Compression test (8.2 and 23.1) [14,15], however the sample size was small in one study [14] and no confidence intervals were reported in either of the studies. Traditional ACJ tests resulted in only a marginal increase in post-test probability for a PAR (13% to 23%) in the current study, with the pre-test probability (14%) contained within the PPV 95% confidence interval of all traditional tests.

When the four traditional ACJ tests were combined, the 95% confidence intervals for the positive and negative likelihood ratios all spanned 1.0 and do not provide sufficient confidence for accurate diagnosis. Although combinations of four positive tests improved the post-test probability to 50%, the confidence interval (0.10, 0.91) included the pre-test probability of 14% limiting confidence in an improvement in the ability to identify ACJ pain.

These results suggest that widespread acceptance of the Active Compression/O’Brien’s, and other traditional ACJ tests as valuable diagnostic tests for ACJ pain in primary health care settings may have been premature. Acceptance of these tests as valuable diagnostic tools for ACJ pain in clinical practice appears to have been based upon the results of a small number of studies that reported high sensitivity or specificity values for injection-confirmed ACJ pain (Table 1) [13-16]. While sensitivity and specificity provide a useful indication of probability of a positive or negative clinical test result in those known to have a condition (e.g. ACJ pain), likelihood ratios are considered more powerful indicators of disease status in individual patients when their condition is unknown which is more reflective of clinical practice [17]. In previous studies, the positive and negative likelihood ratios appear to provide little supporting evidence of a meaningful change in the probability of ACJ pain or pathology despite high reported point estimates of sensitivity and specificity. For instance, despite a reported specificity of 90% for the Active Compression/O’Brien’s test, the LR+ was only 1.6 (confidence intervals, and contingency table not reported) [15] and localised ACJ tenderness was reported to be 96% sensitive, however the LR- was 0.40 (confidence intervals and contingency table not reported) [13] providing little confidence in the ability to rule-out ACJ pain when tenderness was not elicited. This highlights the importance of interpreting all reported diagnostic values along with estimates of precision prior to accepting tests into clinical practice.

In contrast to previous results in secondary and tertiary care populations [13,14,16], localised ACJ tenderness to palpation and the cross-body adduction test were not strongly associated with a PAR to ACJ diagnostic block in this primary care cohort. In addition to the issues surrounding interpretation of diagnostic test values, these findings may relate to differences in disease severity in our study compared with previous work [19]. In previous studies investigating the accuracy of clinical tests for ACJ pain, patients were recruited from surgical waiting lists (patients awaiting distal clavicle excision) [14] or from specialist shoulder centres [13] in which the severity and duration of ACJ disease are likely to differ considerably from primary care patients presenting for the first time with a new episode of shoulder pain (inclusion criteria in the current study). Pain provocation during the cross-body adduction test and the ability to provoke symptoms of ACJ pain with manual palpation may be influenced by the type and severity of pathology and corresponding levels of mechanical and chemical sensitisation providing a possible explanation for this finding. Disease severity is thus likely to limit the generalisability of diagnostic accuracy results obtained in secondary and tertiary care settings, to primary care patients, and vice versa [13,14].

### Limitations

There were some limitations to consider in the current study. Despite previous reliability testing for physical examination tests used in this study [44], the interobserver reliability of the history variables and observation of ACJ swelling or thickening requires further evaluation. Furthermore, the use of strict cut-off criteria for a PAR may eliminate cases where the result may still produce a clinically meaningful outcome and on-going analyses will be conducted in which various anaesthetic response levels will be used as outcome variables. The number of PAR cases was also relatively small and validation of these results in a larger sample is required.

## Conclusions

In conclusion, traditional ACJ tests were of limited diagnostic value for identifying the ACJ as the predominant source of pain in a cohort of primary care patients. Combinations of several other history and physical examination findings including pain referral patterns, mechanism of pain onset and passive range of motion tests appear to be of more diagnostic value for identifying painful ACJ conditions in a sample of primary care patients with predominantly non-traumatic shoulder pain. This may aid early and accurate identification of symptomatic ACJ pathology in primary health care enabling more efficient application of appropriate treatment interventions.

## Abbreviations

ACJ: Acromioclavicular joint; LR+: Positive likelihood ratio; LR: Negative likelihood ratio; PAR: Positive anaesthetic response; CI: Confidence interval; MRI: Magnetic resonance imaging; STARD: Standards for; SPADI: Shoulder Pain and Disability Index; FABQ: Fear Avoidance Beliefs Questionnaire; ROM: Range of motion; VAS: Visual analogue scale; ROC: Receiver operator curve; PPV: Positive predictive value

## Competing interests

The authors declare that they have no competing interests.

## Authors’ contributions

All authors were involved with conception and design of the study. AC performed all the clinical examination and pre-and post-injection clinical tests, collected and managed all data, carried out the preliminary analysis and drafted the manuscript. ML contributed to study design and methodology. ML, WH and PM contributed to methodological development, interpretation of data and critical appraisal of the manuscript for academic and clinical content. All authors read and approved the final manuscript.

## Pre-publication history

The pre-publication history for this paper can be accessed here:

http://www.biomedcentral.com/1471-2474/14/156/prepub

## Supplementary Material

Additional file 1 **Clinical examination variables.** A summary of all the self-report and clinical examination variables recorded and used in the data analysis. Click here for file
