# Supplementary material for: Shoulder pain in primary care: diagnostic accuracy of clinical examination tests for non-traumatic acromioclavicular joint pain
Source: BMC Musculoskelet Disord. 2013 May 1;14:156. doi: 10.1186/1471-2474-14-156 (PMC3646690; doi:10.1186/1471-2474-14-156)
Supplement: Additional file 1 — Clinical examination variables. A summary of all the self-report and clinical examination variables recorded and used in the data analysis. [file 1471-2474-14-156-S1.pdf]

Additional File 1. Clinical examination variables

Clinical examination component	Response outcome
**Self Report Questionnaires**	
SF-8 Health Survey	% (dichotomised by median)
Shoulder Pain and Disability Index (SPADI)	% (dichotomised by median)
Fear Avoidance Beliefs Questionnaire (FABQ)	% (dichotomised by median)
**Patient History**	
Medical screening questionnaire:<br>Medical conditions<br>Smoking status	Dichotomous responses
Symptom chart:<br>Pain location<br>Pain intensity<br>Nature of pain<br>Night pain disturbs sleep<br>Able to sleep on affected side<br>Aggravating activities<br>Easing activities	 Categorical (anterior, posterior, superior, lateral, deep)<br>100mm VAS<br>Constant/intermittent<br>Yes/No<br>Yes/No<br>List<br>List
History of current episode:<br>Date of onset<br>Mechanism of pain onset<br>Previous history of shoulder pain<br>Previous episodes fully resolved<br>Family history of shoulder pain	 Date<br>Traumatic, strain, repetitive, insidious<br>Yes/No<br>Yes/No<br>Yes/No
**Physical Examination**	
Height/weight	Standard SI units
Observation:<br>Supraspinatus atrophy<br>Infraspinatus atrophy<br>ACJ swelling/thickening	Present/absent
Cervical spine testing:<br>Reproduction of shoulder symptoms<br>Production of cervical spine symptoms	Yes/No
Shoulder active ROM:<br>[a]Elevation (flexion)<br>Hand-behind-back<br>Hand-behind-head	 ROM (^{o}); symptom responses: Yes/No<br>ROM (cm); symptom responses: Yes/No<br>ROM (cm); symptom responses: Yes/No
[a] *Shoulder passive ROM:*<br>Glenohumeral abduction<br>External rotation (0° abd)<br>External rotation (90° abd)<br>Internal rotation (90° abd)<br>Cross-body adduction	ROM (^{o}); symptom responses: Yes/No
[a]*Shoulder resisted tests:*<br>Abduction<br>External rotation<br>Internal rotation	Peak muscle force (kg); symptom responses: Yes/No
Scapuloclavicular tests:<br>Elevation<br>Depression<br>Protraction<br>Retraction	Symptom responses: Yes/No
Orthopaedic special tests:<br>Painful arc abduction	Positive/Negative according to original test criteria.

Hawkins-Kennedy test<br>Empty can test<br>Drop-arm test<br>External rotation lag sign<br>Belly press test<br>Active compression test<br>Speeds test<br>Apprehension/Relocation test	
Palpation:<br>Acromioclavicular joint<br>Greater tuberosity (supraspinatus insertion)<br>Lesser tuberosity (subscapularis insertion)<br>Long head of biceps tendon	Typical pain reproduced Yes/No

Abbreviations: VAS, visual analogue scale; ACJ, acromioclavicular joint; ROM, range of motion
